# Trends in youth e-cigarette and cigarette use between 2013 and 2019: insights from repeat cross-sectional data from the COMPASS study

**DOI:** 10.17269/s41997-020-00389-0

**Published:** 2020-08-17

**Authors:** Adam G. Cole, Sarah Aleyan, Kate Battista, Scott T. Leatherdale

**Affiliations:** 1 Faculty of Health Sciences, University of Ontario Institute of Technology, 2000 Simcoe Street North, Oshawa, Ontario L1G 0C5 Canada; 2 School of Public Health and Health Systems, University of Waterloo, Waterloo, Ontario Canada; 3 Institute of Psychiatry, Psychology & Neuroscience, King’s College London, London, UK

**Keywords:** E-cigarette, Cigarette, Smoking, Adolescent, Youth, Trends, Cigarettes électroniques, cigarettes, fumer, adolescent, jeunes, tendances

## Abstract

**Objectives:**

E-cigarettes are an increasingly popular product among youth in Canada. However, there is a lack of long-term data presenting trends in use. As such, the objective of this study was to examine trends in e-cigarette and cigarette use across various demographic characteristics between 2013 and 2019 among a large sample of secondary school youth in Canada.

**Methods:**

Using repeat cross-sectional data from a non-probability sample of students in grades 9 to 12, this study explored trends in the prevalence of ever and current e-cigarette use and cigarette smoking between 2013–2014 and 2018–2019 in British Columbia, Alberta, Ontario, and Quebec. Trends in ever and current e-cigarette use and cigarette smoking were studied across demographic variables among students in Ontario.

**Results:**

The prevalence of e-cigarette ever and current use was variable across province and increased over time, particularly between 2016–2017 and 2018–2019. In contrast, the prevalence of current cigarette smoking was relatively stable over the study period, decreasing significantly in Alberta and Ontario between 2017–2018 and 2018–2019. In Ontario, the prevalence of ever and current e-cigarette use increased among all grades, both genders, and all ethnicities.

**Conclusion:**

Consistent with data from the United States, the prevalence of e-cigarette use among our large sample of Canadian youth has increased substantially in a short period of time. Surveillance systems should continue to monitor the prevalence of tobacco use among youth. Additional interventions may be necessary to curb e-cigarette use among Canadian youth.

**Electronic supplementary material:**

The online version of this article (10.17269/s41997-020-00389-0) contains supplementary material, which is available to authorized users.

## Résumé

**Objectifs:**

Les cigarettes électroniques sont de plus en plus populaires auprès des jeunes au Canada. Il manque cependant de données à long terme sur les tendances de leur usage. C’est pourquoi nous avons cherché à examiner les tendances de l’usage des cigarettes électroniques et des cigarettes selon diverses caractéristiques démographiques entre 2013 et 2019 dans un vaste échantillon de jeunes fréquentant l’école secondaire au Canada.

**Méthode:**

À l’aide de données transversales répétées d’un échantillon non probabiliste d’élèves de la 9^e^ à la 12^e^ année, l’étude a exploré les tendances de la prévalence de l’usage antérieur et actuel des cigarettes électroniques et des cigarettes entre 2013-2014 et 2018-2019 en Colombie-Britannique, en Alberta, en Ontario et au Québec. Les tendances de l’usage antérieur et actuel des cigarettes électroniques et des cigarettes ont été étudiées pour diverses variables démographiques chez les élèves de l’Ontario.

**Résultats:**

La prévalence de l’usage antérieur et actuel des cigarettes électroniques varie selon la province et a augmenté avec le temps, particulièrement entre 2016-2017 et 2018-2019. Par contre, la prévalence de l’usage actuel des cigarettes a été relativement stable sur la période de l’étude et a sensiblement diminué en Alberta et en Ontario entre 2017-2018 et 2018-2019. En Ontario, la prévalence de l’usage antérieur et actuel des cigarettes électroniques a augmenté chez les élèves de toutes les années, des deux sexes et de toutes les origines ethniques.

**Conclusion:**

Comme pour les données des États-Unis, la prévalence de l’usage des cigarettes électroniques dans notre vaste échantillon de jeunes Canadiens a considérablement augmenté sur une courte période. Les systèmes de surveillance devraient continuer à surveiller la prévalence du tabagisme chez les jeunes. Des interventions supplémentaires pourraient être nécessaires pour freiner l’usage des cigarettes électroniques chez les jeunes Canadiens.

## Introduction

Declines in combustible cigarette use have been observed over the last two decades both nationally and globally (Arrazola et al. 2015; Reid et al. 2017). In contrast, the prevalence of use of e-cigarettes, a recent addition to the nicotine market, has increased dramatically over the last few years (Arrazola et al. 2015; Montreuil et al. 2017), with Juul becoming one of the most popular brands (Huang et al. 2019). Most trend data for youth e-cigarette use are from the United States (USA), where tobacco and e-cigarette policies are different from those in Canada. For example, up until May 2018, e-cigarettes containing nicotine were not legally available for sale in Canada, while these types of e-cigarettes were widely available in the USA. Furthermore, while both the USA and Canada have minimum legal age restrictions for purchasing e-cigarettes, some states have recently raised the minimum legal age to 21 in order to make it more difficult for youth to access these products, while the minimum legal age in Canada remains 18 or 19 years (depending on the province (Tobacco and Vaping Products Act 2018)). These variations in e-cigarette policies between countries can influence e-cigarette use rates across the population.

Data from two large population studies of high school students in the USA indicate that the prevalence of e-cigarette use increased dramatically between 2017 and 2019, sparking alarm of an epidemic (CDC 2019; Miech et al. 2019). A single study of a small, random sample of adolescents 16–19 years old from Canada also suggests an increase in the prevalence of e-cigarette use (Hammond et al. 2019). The popularity of e-cigarettes has sparked heated debate within the public health community for how to balance their potential public health impact. Some have advocated for the use of e-cigarettes as a potential smoking cessation aid and harm reduction tool among established adult smokers (Flahault and Etter 2014). Others have raised concerns that e-cigarette use may result in the re-normalization of smoking behaviours (Stanwick 2015) and lead to combustible tobacco use, particularly among youth (Aleyan et al. 2018; Soneji et al. 2017).

Currently, there is a dearth of literature examining long-term trends in adolescent e-cigarette use within the Canadian context, where the e-cigarette regulatory environment and market have differed from many other jurisdictions. Until recently, Canadian laws restricted the sale, marketing, and advertising of nicotine-containing e-cigarettes, which would impact the sale of certain brands (e.g., Juul). As a result, it is possible that the more restrictive policy environment in Canada may have had a differential impact on trends in youth e-cigarette use (i.e., smaller increase in prevalence relative to the USA). Variations in provincial policies targeting e-cigarette use may also impact trends among youth. There are also limited data comparing trends in the prevalence of e-cigarette use across various demographic characteristics; different subgroups of youth may have experienced greater changes in the prevalence of e-cigarette use relative to others and could be a target for future interventions. Finally, given evidence indicating associations between e-cigarette use and cigarette smoking (Soneji et al. 2017), it is important to monitor changes in cigarette smoking to see whether population-level changes in e-cigarette use impact the population-level prevalence of cigarette smoking. The objective of this study was to examine trends in e-cigarette and cigarette use across various demographic characteristics between 2013 and 2019 among a large, non-probability sample of secondary school students in Canada.

## Methods

### Sample selection

This study used repeat cross-sectional data from a non-probability sample of students in grades 9 to 12 in four Canadian provinces beginning in 2013–2014 (the first year e-cigarette use was measured in the host study) and up to and including 2018–2019 of the COMPASS study. COMPASS is a Canadian Institutes of Health Research (CIHR)-funded 9-year (2012–2021) school-based, prospective cohort study designed to evaluate the impact of changes to programs, policies, and the built environment on multiple youth health behaviours and outcomes over time (Leatherdale et al. 2014). Between 2013–2014 and 2018–2019, public and private schools that used active-information passive-consent parental permission protocols (passive consent) were purposefully sampled from Ontario and Alberta (Thompson-Haile and Leatherdale 2013). Following the receipt of additional funding from Health Canada, the project was expanded in 2016–2017 using similar recruitment methods to include secondary schools in British Columbia and Quebec. Additional details regarding the recruitment methods of the COMPASS study can be found in print (Leatherdale et al. 2014) or online (www.compass.uwaterloo.ca). The University of Waterloo Office of Research Ethics and participating school board ethics committees approved all procedures.

E-cigarette use and cigarette smoking trend data are presented for all four provinces. However, trends in e-cigarette use and cigarette smoking according to demographic characteristics are only presented using data from Ontario where the sample size of students and schools was larger (Supplementary Table 1). Over the course of the study, between 29,754 and 41,048 students from 61 to 79 schools participated from across Ontario. Across years, student participation rates were generally high (> 75%) and participant demographics were similar. At every year, approximately half of students were female, more than three quarters were white, and slightly more than half were in grades 9 and 10 (Supplementary Table 2).

### Measures

Data were collected annually from students using the COMPASS questionnaire (Cq), a paper-based survey completed during class time between October and June every year. In addition to information on socio-demographic characteristics, the Cq collects information on smoking and e-cigarette use behaviours using items consistent with other school-based research in Canada and with demonstrated reliability and validity for current smoking (Wong et al. 2012).

#### E-cigarette use behaviours

In every year, students were asked to respond to a multi-item question, “In the past 30 days, did you use any of the following? (Mark all that apply),” followed by a list of tobacco products other than cigarettes which included e-cigarettes (electronic cigarettes that look like cigarettes/cigars, but produce vapour instead of smoke). Beginning in 2015–2016, students were asked to respond to the question, “Have you ever tried an electronic cigarette, also known as an e-cigarette?” A derived variable was created by combining responses to these two questions. Those who never used e-cigarettes were classified as *never users*, while those who reported using e-cigarettes in the last 30 days were classified as *current users*. Those who reported ever using e-cigarettes but not in the last 30 days were classified as *non-current users*.

#### Smoking susceptibility

Consistent with prior research, smoking susceptibility among never-smoking students was assessed through the use of three previously validated measures that asked respondents if they might try smoking in the future, if they would smoke if their best friend offered a cigarette, and if they might smoke a cigarette in the next year (Cole et al. 2017a; Pierce et al. 1996). Students who responded “definitely not” to all three questions were classified as *non-susceptible never smokers*; all other students were classified as *susceptible never smokers*.

#### Cigarette smoking behaviours

Two questions assessed cigarette smoking status: “Have you ever tried cigarette smoking, even a few puffs?” and “On how many of the last 30 days did you smoke one or more cigarettes?” To be consistent with the definition of e-cigarette use, a derived variable was created by combining responses to these two questions. Those who never smoked cigarettes were classified as *never smokers* (and could be classified as susceptible or non-susceptible as described previously), while those who reported smoking cigarettes within the last 30 days were classified as *current smokers.* Those who ever smoked cigarettes but not in the last 30 days were classified as *non-current smokers.*

#### Socio-demographic characteristics

Students self-reported their gender (male or female), school grade (9, 10, 11, or 12), and ethnicity (white, Black, Asian, Latin American/Hispanic, other/mixed).

### Analysis

Statistical analyses were conducted using SAS 9.4. We first examined the prevalence of e-cigarette use and cigarette smoking for each year in each province. Because there were multiple waves of data available and there was a larger sample of students and schools (see Supplementary Table 1), trends in e-cigarette use and cigarette smoking were examined by gender, grade, ethnicity, and cigarette smoking/e-cigarette use status only among students in Ontario. Logistic regression models using the GENMOD procedure confirmed overall trends in e-cigarette and cigarette use while controlling for grade, gender, ethnicity, cigarette smoking/e-cigarette use status, and school-level clustering. Only 2.3% of data were excluded due to missing outcomes or covariates. Statistical significance was set at *p* < 0.05.

## Results

**Fig. 1:**
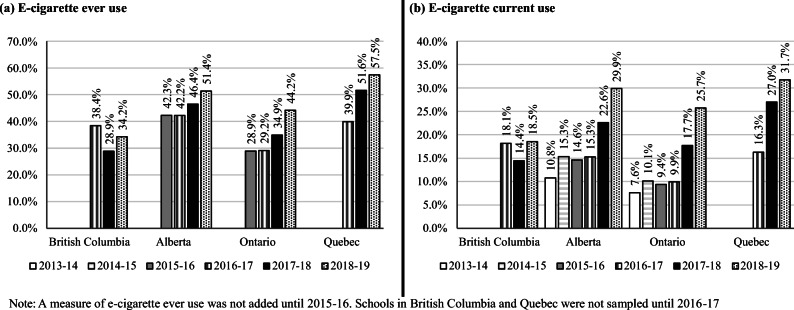
Prevalence of e-cigarette **(a)** ever use and **(b)** current use, by province and year, 2013–2019 COMPASS study

**Fig. 2:**
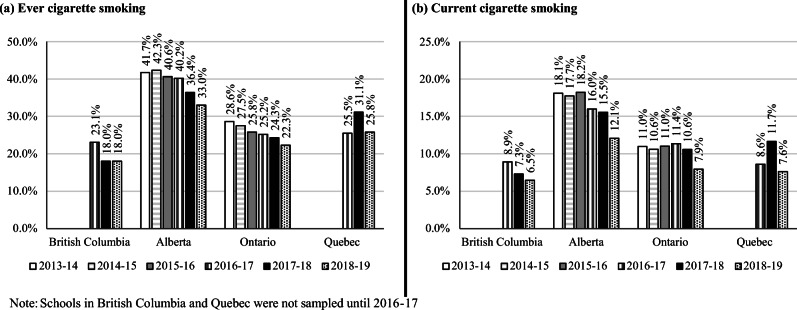
Prevalence of (**a)** ever cigarette smoking and **(b)** current cigarette smoking, by province and year, 2013–2019 COMPASS study

Figure 1 presents the prevalence of e-cigarette ever and current use by province. As evident in the figure, the prevalence of e-cigarette ever and current use was variable across province and increased over time. Most notably, the prevalence of e-cigarette current use increased significantly and roughly doubled between 2016–2017 and 2018–2019 in Alberta, Ontario, and Quebec (all *p* < 0.0001). Figure 2 presents the prevalence of cigarette ever and current smoking by province. Similar to Fig. 1, the prevalence of cigarette ever and current smoking was variable across province. Although the prevalence of current cigarette smoking was fairly consistent in Alberta and Ontario between 2013–2014 and 2015–2016, the prevalence was significantly lower in 2018–2019 relative to 2017–2018 (both *p* < 0.0001).

### Trends in e-cigarette use in Ontario

In the Ontario sample between 2013–2014 and 2018–2019, the prevalence of e-cigarette ever and current use increased among all grades, both genders, and all ethnicities (Table 1). In Ontario, 7.6% of students reported currently using e-cigarettes in 2013–2014, but by 2018–2019, 25.7% of students reported currently using e-cigarettes (an increase of 238%). Generally, at all time-points e-cigarette use was highest among males and grade 12 students, and lowest among females, grade 9 students, and Asian students. For most groups, the prevalence of e-cigarette ever use increased first between 2016–2017 and 2017–2018, and again between 2017–2018 and 2018–2019. Similarly, the prevalence of e-cigarette current use first increased between 2013–2014 and 2014–2015, then increased between 2016–2017 and 2017–2018, and increased again between 2017–2018 and 2018–2019. When stratified by cigarette smoking status, the prevalence of e-cigarette ever and current use increased among all groups. Overall, the logistic regression models indicate that, relative to 2015–2016, students had higher odds of ever using e-cigarettes in 2017–2018 (OR = 1.54, 95% CI 1.43–1.67) and 2018–2019 (OR = 2.81, 95% CI 2.60–3.03). Similarly, relative to 2013–2014, students had higher odds of currently using e-cigarettes in 2017–2018 (OR = 3.20, 95% CI 2.84–3.61) and 2018–2019 (OR = 6.33, 95% CI 5.67–7.08).

**Table 1:** Prevalence of e-cigarette ever use and current use among secondary students in Ontario according to socio-demographic factors and cigarette smoking status, by year, 2013–2019 COMPASS study

	2013–2014 (%)	2014–2015 (%)	2015–2016 (%)	2016–2017 (%)	2017–2018 (%)	2018–2019 (%)
E-cigarette ever use^a^	–	–	28.9	29.2	34.9	44.2
Grade						
9	–	–	20.0	18.4	24.2	33.0
10	–	–	27.2	29.0	33.3	43.8
11	–	–	32.7	34.3	41.2	49.3
12	–	–	37.9	37.3	43.8	54.1
Gender						
Female	–	–	22.0	23.1	29.7	41.4
Male	–	–	35.4	35.1	40.0	46.9
Ethnicity						
White	–	–	28.9	29.6	36.3	46.3
Black	–	–	34.5	29.9	30.8	37.6
Asian	–	–	20.4	17.8	21.7	29.0
Latin American/Hispanic	–	–	30.4	30.4	34.1	44.4
Other/mixed^b^	–	–	32.0	33.0	37.0	44.5
Cigarette smoking status						
Never smoked, non-susceptible	–	–	11.5	11.9	16.7	26.5
Never smoked, susceptible	–	–	26.7	26.0	34.5	47.0
Non-current smoker	–	–	57.9	62.1	70.2	82.4
Currently smokes	–	–	77.2	74.9	80.9	88.8
E-cigarette current use	7.6	10.1	9.4	9.9	17.7	25.7
Grade						
9	5.3	7.1	6.6	6.8	12.0	18.5
10	8.0	10.6	8.1	9.9	16.8	26.0
11	9.2	11.4	10.8	12.1	21.3	29.3
12	8.3	11.8	12.7	11.6	22.0	31.2
Gender						
Female	5.2	7.2	5.9	6.6	13.9	23.4
Male	10.0	13.0	12.6	13.2	21.4	28.0
Ethnicity						
White	7.2	9.5	9.1	10.0	18.8	27.7
Black	12.9	15.5	14.8	12.3	15.4	19.8
Asian	6.0	7.5	7.0	5.5	9.2	13.3
Latin American/Hispanic	9.2	12.7	9.1	9.9	15.6	24.2
Other/mixed^b^	10.3	14.8	10.8	11.3	16.9	24.8
Cigarette smoking status						
Never smoked, non-susceptible	1.7	2.5	2.1	2.7	6.9	12.5
Never smoked, susceptible	5.3	7.8	6.7	7.0	17.0	26.4
Non-current smoker	11.6	16.0	15.4	17.0	31.6	52.1
Currently smokes	32.7	42.2	41.1	40.9	55.1	68.0

^a^A measure of e-cigarette ever use was not added until 2015–2016

^b^Students who identified as “off-reserve Aboriginal” are also included in this category

### Trends in cigarette smoking in Ontario

In the Ontario sample between 2013–2014 and 2017–2018, the prevalence of ever and current cigarette smoking was relatively consistent across all grades, genders, and ethnicities (Table 2); the prevalence of ever and current cigarette smoking decreased significantly between 2017–2018 and 2018–2019 (both *p* < 0.0001). In Ontario in 2013–2014, 11.0% of students reported currently smoking cigarettes; by 2018–2019, 7.9% of students reported currently smoking cigarettes (a decrease of 28%). Generally, at all time-points cigarette smoking was highest among males and grade 12 students, and lowest among females, grade 9 students, and Asian students. When stratified by e-cigarette use status, the prevalence of cigarette smoking decreased among all groups, particularly between 2017–2018 and 2018–2019. Overall, the logistic regression models indicate that relative to 2013–2014, students had lower odds of ever (OR = 0.27, 95% CI 0.25–0.30) and currently (OR = 0.29, 95% CI 0.26–0.33) smoking cigarettes in 2018–2019.

**Table 2:** Prevalence of ever and current cigarette smoking among secondary school students in Ontario according to socio-demographic factors and e-cigarette use status, by year, 2013–2019 COMPASS study

	2013–2014 (%)	2014–2015 (%)	2015–2016 (%)	2016–2017 (%)	2017–2018 (%)	2018–2019 (%)
Ever smoked cigarettes	28.6	27.5	25.8	25.2	24.3	22.3
Grade						
9	16.5	15.5	14.5	13.4	13.0	12.3
10	25.9	24.6	21.9	23.8	20.8	20.2
11	33.8	33.7	30.7	29.5	30.8	26.3
12	40.7	39.2	39.2	37.5	36.2	34.3
Gender						
Female	26.3	25.8	23.4	23.4	22.6	20.9
Male	30.9	29.1	28.1	27.0	26.0	23.7
Ethnicity						
White	27.6	26.4	25.0	24.6	24.0	21.9
Black	33.1	32.9	31.4	27.0	23.3	21.2
Asian	19.2	19.1	17.7	15.8	14.8	14.6
Hispanic	34.7	32.7	31.5	25.9	23.6	22.2
Other/mixed^b^	43.3	40.6	36.6	37.9	36.7	34.7
E-cigarette use status						
Never used	–	–	12.3	11.4	9.4	6.1
Non-current user^a^	24.9	22.6	52.5	52.7	46.7	32.6
Currently uses	73.8	70.9	72.6	70.4	57.5	50.0
Current cigarette smoking	11.0	10.6	11.0	11.4	10.6	7.9
Grade						
9	5.8	5.8	5.8	6.0	5.6	4.7
10	9.9	9.5	9.3	10.9	9.3	6.8
11	13.3	12.7	12.9	13.2	13.7	9.0
12	16.0	15.7	17.6	16.9	15.3	12.6
Gender						
Female	9.1	9.0	9.0	9.8	8.9	6.8
Male	12.8	12.1	12.9	12.9	12.2	9.0
Ethnicity						
White	10.3	9.7	10.2	10.7	10.2	7.3
Black	15.7	15.8	17.3	14.5	11.8	10.4
Asian	6.4	6.1	6.4	6.7	5.3	3.9
Hispanic	11.5	13.2	12.7	11.7	7.4	6.3
Other/mixed^b^	19.1	19.8	18.3	19.5	19.3	16.2
E-cigarette use status						
Never used	–	–	3.5	4.0	3.1	1.6
Non-current user^a^	8.0	6.8	20.3	20.1	15.9	8.9
Currently uses	47.0	44.3	48.3	46.7	33.0	20.9

^a^Ever use of e-cigarettes was not assessed prior to 2015; non-current users include those who have never used e-cigarettes and those who did not use e-cigarettes in the last 30 days

^b^Students who identified as “off-reserve Aboriginal” are also included in this category

## Discussion

These are some of the most comprehensive data showing changes in e-cigarette use and cigarette smoking prevalence in Canada over the last 6 years. According to these data, current e-cigarette use increased substantially over three time-points: first between 2013–2014 and 2014–2015, then between 2016–2017 and 2017–2018, and again between 2017–2018 and 2018–2019. Within the Ontario sample, the trend was similar across grade, gender, ethnicity, and cigarette smoking status. The data also indicate that, over the same time period, ever and current cigarette smoking were relatively stable, although a decrease in the prevalence of cigarette smoking was apparent in the most recent data collection wave. The trend was also similar across grade, gender, ethnicity, and e-cigarette use status.

The large relative increase in the prevalence of e-cigarette use between 2016–2017 and 2018–2019 is particularly noteworthy as it marks a period when e-cigarette use is more popular than cigarette smoking. In fact, in British Columbia, Ontario, and Quebec, approximately three times as many youth report currently using e-cigarettes as cigarettes. A shift from traditional tobacco products to vaping products has been noted in other jurisdictions (CDC 2019). To help control the rapid increase in the popularity of e-cigarettes, provincial and national governments have expanded smoke-free space regulations to include e-cigarettes (including banning the use of e-cigarettes on elementary and secondary school campuses (Smoke-Free Ontario Act 2017)) and created minimum legal age requirements for purchasing e-cigarettes (Tobacco and Vaping Products Act 2018). Despite these controls, it is apparent that many youth are able to obtain and continue to use e-cigarettes. Additional data about where students access e-cigarettes and the types of e-cigarettes, the nicotine content, and the flavours used by Canadian youth may be needed to inform the development of novel policies, programs, and resources that prevent youth from accessing and using these products.

The dramatic and rapid increase in the prevalence of e-cigarette use mirrors a similar increase that was observed in the USA (CDC 2019; Miech et al. 2019) and other recent Canadian data (Hammond et al. 2019). During this period, the e-cigarette market in the USA shifted drastically as Juul became the dominant brand (Huang et al. 2019; King et al. 2018). As of May 2018, the Tobacco and Vaping Products Act allows for the sale and promotion of nicotine-containing e-cigarettes (except for lifestyle advertising and advertising considered appealing to youth) (Tobacco and Vaping Products Act 2018). The current data indicate that the prevalence of e-cigarette use among youth began to increase before nicotine-containing e-cigarettes were legally available for sale in Canada (May 2018) and continued to rise after they were introduced into the Canadian market; the Act is likely to have resulted in an additional shift in the e-cigarette market in Canada (i.e., increased availability of nicotine-containing e-cigarettes). Future work should continue to examine trends in e-cigarette use among youth, young adult, and adult populations in Canada. In particular, it will be important to identify whether youth are using nicotine-containing e-cigarettes and whether there are shifts in device preference as brands are allowed to advertise their product.

In contrast with an earlier study (Hammond et al. 2019), our results indicate that youth cigarette smoking decreased significantly between 2017–2018 and 2018–2019. Although the current study is not nationally or provincially representative, the large, school-based sample and the purposeful use of passive consent procedures (given the focus on substance use in the COMPASS study) dramatically limit bias that results from student non-response and non-participation common in active consent studies of youth substance use (Courser et al. 2009; Pokorny et al. 2001). Previous research using active consent protocols (e.g., Hammond et al. 2019), despite being representatively sampled, will underestimate substance use among youth populations. As such, the different sampling and recruitment procedures employed and differences in the timing of data collections may account for the differences observed. Consistent with other data from the USA (Dutra and Glantz 2017; Levy et al. 2018), it appears as though the increase in youth e-cigarette use has not led to an increase in youth smoking. The availability of flavours of e-cigarettes may have increased their appeal over traditional cigarettes (Zhu et al. 2014); for example, within Ontario as of January 1, 2017, menthol-flavoured cigarettes were banned (Chaiton et al. 2018), leaving e-cigarettes as one of the few flavoured tobacco products available. Other evidence suggests that promotion on social media may have also contributed to the increased appeal of e-cigarettes over traditional cigarettes (Allem et al. 2018; Laestadius et al. 2018).

A variety of intervention strategies may help to reduce the availability and appeal of e-cigarettes among youth. For example, given that the appealing flavours are an important reason for youth use of e-cigarettes (Meernik et al. 2019), limiting the number and types of e-cigarette flavours available may help to reduce their appeal and use among youth. Data from clinical trials suggest that lowering the nicotine content of cigarettes reduces cigarette consumption among adults (Benowitz et al. 2012; Donny et al. 2015); it is possible that a similar approach to limiting the amount of nicotine that is permitted in pods and e-liquid may help to reduce e-cigarette use among youth. Similar to tobacco control measures, restricting e-cigarette advertisements and promotions may also help to reduce the appeal and use of e-cigarettes among youth, given the high rates of ad exposure (Cho et al. 2019). Finally, given that the school environment is a unique setting of influence where youth spend a significant amount of time and are influenced by programs, policies, and peers (Cole et al. 2017b; Lovato et al. 2010; Murnaghan et al. 2007; Thomas et al. 2015), school-based prevention and cessation interventions could also be a part of a comprehensive strategy.

Limitations to this study include the use of a non-probability, school-based sample which may not represent all youth in each province or within Canada, self-report tobacco use data, and the later addition of a measure of e-cigarette ever use. Additionally, the use of a “select all that apply” question to measure e-cigarette use rather than a stand-alone question may underestimate the prevalence of use (Delnevo et al. 2017). Furthermore, we were unable to assess whether students were using nicotine-containing e-cigarettes. Finally, as a result of the changing language used by youth to refer to e-cigarette devices (e.g., vaping, Juuling, Vyping), our study may underestimate the prevalence of e-cigarette use.

## Conclusion

Consistent with data from the USA (CDC 2019; Miech et al. 2019), the prevalence of e-cigarette use among youth in Canada has increased substantially in a short period of time; however, a decrease in the prevalence of cigarette smoking has lagged. Increases in e-cigarette use were apparent across demographic groups. Surveillance systems should continue to monitor the prevalence of tobacco and e-cigarette use among youth populations. Additional regulations and interventions may be necessary to curb e-cigarette use among Canadian youth.

## Electronic supplementary material

ESM 1 (DOCX 13 kb)

ESM 2 (DOCX 13.4 kb)
