# Correction to: Trends in youth e-cigarette and cigarette use between 2013 and 2019: insights from repeat cross-sectional data from the COMPASS study

**DOI:** 10.17269/s41997-020-00441-z

**Published:** 2020-11-10

**Authors:** Adam G. Cole, Sarah Aleyan, Kate Battista, Scott T. Leatherdale

**Affiliations:** 1 Faculty of Health Sciences, University of Ontario Institute of Technology, 2000 Simcoe Street North, Oshawa, Ontario L1G 0C5 Canada; 2 School of Public Health and Health Systems, University of Waterloo, Waterloo, Ontario Canada; 3 Institute of Psychiatry, Psychology & Neuroscience, King’s College, London, London, UK

**Correction to: Canadian Journal of Public Health**

10.17269/s41997-020-00389-0

This article was updated to include additional funding information in the Acknowledgements.

