# Supplementary material for: Trends in youth e-cigarette and cigarette use between 2013 and 2019: insights from repeat cross-sectional data from the COMPASS study
Source: Can J Public Health. 2020 Aug 17;112(1):60–9. doi: 10.17269/s41997-020-00389-0 (PMC7851234; doi:10.17269/s41997-020-00389-0)
Supplement: Supplementary file 1 — (DOCX 13 kb) [file 41997_2020_389_MOESM1_ESM.docx]

**Supplementary Table 1. Number of participating students and schools, by province and year, 2013–2019 COMPASS study**

|  | 2013–2014 | | 2014–2015 | | 2015–2016 | | 2016–2017 | | 2017–2018 | | 2018–2019 | |
| --- | --- | --- | --- | --- | --- | --- | --- | --- | --- | --- | --- | --- |
|  | # schools | # students | # schools | # students | # schools | # students | # schools | # students | # schools | # students | # schools | # students |
| Total | 89 | 44,572 | 87 | 41,669 | 81 | 39,671 | 93 | 42,950 | 122 | 56,412 | 136 | 58,845 |
| British Columbia | - | - | - | - | - | - | 5 | 3,496 | 16 | 12,123 | 15 | 10,025 |
| Alberta | 10 | 3,524 | 9 | 3,292 | 9 | 3,264 | 9 | 2,903 | 8 | 3,178 | 8 | 3,213 |
| Ontario | 79 | 41,048 | 78 | 38,377 | 72 | 36,407 | 68 | 33,002 | 61 | 30,806 | 61 | 29,754 |
| Quebec | - | - | - | - | - | - | 11 | 3,549 | 37 | 10,305 | 52 | 15,853 |
