# Supplementary material for: Trends in youth e-cigarette and cigarette use between 2013 and 2019: insights from repeat cross-sectional data from the COMPASS study
Source: Can J Public Health. 2020 Aug 17;112(1):60–9. doi: 10.17269/s41997-020-00389-0 (PMC7851234; doi:10.17269/s41997-020-00389-0)
Supplement: Supplementary file 2 — (DOCX 13.4 kb) [file 41997_2020_389_MOESM2_ESM.docx]

**Supplementary Table 2. Sample characteristics of participating students in Ontario, by year, 2013–2019 COMPASS study**

|  | 2013–2014 | 2014–2015 | 2015–2016 | 2016–2017 | 2017–2018 | 2018–2019 |
| --- | --- | --- | --- | --- | --- | --- |
| Grade |  |  |  |  |  |  |
| 9 | 26.1% | 26.2% | 26.3% | 27.1% | 27.7% | 28.9% |
| 10 | 26.3% | 27.4% | 26.5% | 28.2% | 28.1% | 28.8% |
| 11 | 24.9% | 24.9% | 25.3% | 25.5% | 26.7% | 26.7% |
| 12 | 22.7% | 21.5% | 21.9% | 19.2% | 17.6% | 15.6% |
| Gender |  |  |  |  |  |  |
| Female | 49.4% | 49.4% | 48.4% | 49.7% | 50.2% | 49.9% |
| Male | 50.6% | 50.6% | 51.6% | 50.3% | 49.8% | 50.1% |
| Ethnicity |  |  |  |  |  |  |
| White | 80.5% | 79.4% | 77.9% | 77.8% | 70.8% | 72.8% |
| Black | 4.5% | 5.1% | 5.5% | 4.6% | 4.5% | 4.6% |
| Asian | 5.4% | 5.8% | 6.5% | 7.2% | 14.4% | 12.7% |
| Latin American/ Hispanic | 2.0% | 2.2% | 2.4% | 2.8% | 2.8% | 2.9% |
| Other/mixed ^a^ | 7.5% | 7.5% | 7.6% | 7.7% | 7.6% | 7.0% |

^a^ students who identified as “off-reserve Aboriginal” are also included in this category
